# Comparison of the chemical constituents and anti-Alzheimer’s disease effects of *Uncaria rhynchophylla* and *Uncaria tomentosa*

**DOI:** 10.1186/s13020-021-00514-2

**Published:** 2021-10-27

**Authors:** Qing-Qing Xu, Pang Chui Shaw, Zhen Hu, Wen Yang, Siu-Po Ip, Yan-Fang Xian, Zhi-Xiu Lin

**Affiliations:** 1 School of Chinese Medicine, Faculty of Medicine, The Chinese University of Hong Kong, Shatin, N.T., Hong Kong SAR, People’s Republic of China; 2 School of Life Sciences, Faculty of Science, The Chinese University of Hong Kong, Shatin, N.T., Hong Kong SAR, People’s Republic of China; 3 Li Dak Sum Yip Yio Chin R&D Centre for Chinese Medicine, The Chinese University of Hong Kong, Shatin, N.T., Hong Kong SAR, People’s Republic of China; 4 Hong Kong Institute of Integrative Medicine, The Chinese University of Hong Kong, Hong Kong, Shatin, N.T., Hong Kong SAR, People’s Republic of China

**Keywords:** *Uncaria rhynchophylla*, *Uncaria tomentosa*, Alzheimer’s disease, Streptozotocin, Rats, Akt/GSK3β/Nrf2 pathway

## Abstract

**Background:**

*Uncaria tomentosa*, which has similar chemical constituents with *Uncaria rhynchophylla,* has been reported to alleviate cognitive impairments in Alzheimer’s disease (AD) animal models. This study aimed to compare the chemical constituents and anti-AD effect of the ethanol extracts of *U. tomentosa* (UTE) and *U. rhynchophylla* (URE).

**Methods:**

The high-performance liquid chromatography (HPLC) was used to compare the chemical constituents of UTE and URE. Streptozotocin (STZ) was intracerebroventricularly (ICV) injected into adult male Sprague–Dawley (SD) rats to establish AD model. UTE (400 mg/kg) or URE (400 mg/kg) was administrated intragastrically once daily to the rats for 6 consecutive weeks. Morris water maze (MWM) test was conducted to assess the neurological functions in the STZ-induced AD rats. The brain tissues of the rats were harvested for further biochemical assay.

**Results:**

The MWM test results showed both UTE and URE could significantly improve the learning and memory impairments induced by STZ in rats. Both UTE and URE could significantly inhibit the hyperphosphorylation of tau protein, reduce the elevated levels of pro-inflammatory cytokines (IL-1β, IL-6 and TNF-α), enhance activities of antioxidant enzymes (SOD, CAT and GPx) and increase the protein expression of HO-1. In addition, UTE could decrease the malondialdehyde (MDA) level. Furthermore, both UTE and URE significantly enhanced Akt activation, down regulated the activation of glycogen synthase kinase 3β (GSK-3β), and induced the nuclear translocation of Nrf2 in the STZ-induced AD rats.

**Conclusions:**

UTE and URE contained similar chemical constituents. We found for the first time that both of them could ameliorate cognitive deficits in the STZ-induced AD rats. The underlying molecular mechanism involve suppression of tau hyperphosphorylation, anti-oxidant and anti-neuroinflammation via modulating Akt (Ser473)/GSK3β (Ser9)-mediated Nrf2 activation. These findings amply implicate that both of UTE and URE are worthy of being developed clinically into pharmaceutical treatment for AD.

## Background

Alzheimer’s disease (AD) is a chronic neurodegenerative disease characterized by impaired memory and cognitive functions, and seriously affects one’s daily living. AD is the most common cause of dementia, accounting for 60–70% of all cases of dementia [1]. Approximately 9.8 million people in China and 47 million people worldwide are currently affected by AD or related dementia, and this figure is estimated to triple by 2050 [1, 2]. Moreover, according to a recent report released by World Health Organization, AD became the second biggest killer in high-income countries as well as the seventh leading cause of death worldwide in 2019 [3]. Clearly, AD poses a huge threat to public health, and brings a heavy burden to the patients’ families and societies as a whole.

There is no cure or disease-modifying treatment for AD at present, partly owing to the complex pathogenesis of the disease. Despite its multifactorial etiopathogenesis, two pathological hallmarks in AD brain are believed to contribute to the progression of the disease [4], i.e., neurofibrillary tangles (NFTs) principally consisting of hyperphosphorylated tau protein, and extracellular amyloid plaques mainly composed of aggregated beta-amyloid (Aβ) peptide. Other pathological features, such as neuroinflammation, oxidative stress, synaptic loss and neuronal degeneration, have also been recognized to contribute to the progression of the disease [5]. Mounting evidence suggests that oxidative stress and neuroinflammation play cardinal roles in the early stage of AD as they are interconnected and increase the deposition of both Aβ and tau [6]. However, the consecutive failures of anti-Aβ therapeutics in clinical trials recently indicate that neither Aβ clearance nor other pathological features should be taken as individual targets [6, 7]. The therapeutics now commonly used in clinic are namely acetylcholinesterase inhibitors (AChEIs; e.g., donepezil, rivastigmine and galantamine) and non-competitive *N*-methyl-d-aspartate (NMDA) receptor antagonist (e.g., memantine), but they can only partially relieve the symptoms for a limited period of time [8]. Thus far, these drugs only provide temporary symptomatic relief for AD patients, but cannot delay or stop the underlying pathology [9]. In addition, due to the occurrence of drug resistance and undesirable side effects, these anti-AD drugs have limitations in clinical use [10]. Given the apparent unmet medical need for AD treatment, there clearly exists a need to seek alternative therapeutic approaches.

The etiology of AD is known to be multifactorial, while many traditional medicines and natural products are multi-components and can act on multiple molecular targets to afford the neuroprotective effect in the management of AD [11, 12]. Historically, Chinese herbal medicine (CHM) has been used to treat neurodegenerative disorders and is still widely prescribed in many Eastern Asian countries nowadays [13]. *Uncariae Ramulus Cum Uncis* (also known as Gou-teng in Chinese) is derived from the stem with hook of *Uncaria rhynchophylla* (Uncaria). This herb is one of the most commonly prescribed herbs in Asia, and has been extensively used in Chinese medicine practice to treat a variety of cerebral diseases, such as epilepsy, convulsion, headache and hypertension [14]. It is also incorporated into a number of herbal formulae which are prescribed for the treatment of AD, such as Chotosan (Gouteng-San in Chinese) and Yokukansan (Yigan-San in Chinese) [15, 16]. Our previous study has demonstrated that *U. rhynchophylla* and its major components have potent neuroprotective effects on neurodegenerative diseases, such as AD. Specifically, *U. rhynchophylla* was found to ameliorate cognitive deficits in a mouse model of AD, and the mechanism was related to the inhibition of AChE activity and the enhancement of antioxidant activity [17]. Furthermore, previous studies in our laboratory also revealed that isorhynchophylline (IRN), the major chemical ingredient of *U. rhynchophylla*, played a predominant role in neuroprotection and exerted extensive bioactivities in both in vitro and in vivo models of AD [18–22].

Cat’s claw, whose name comes from the shape of the crooked thorns on its woody vines, is indigenous to the Amazon River basin [23]. It is one of the best-selling herbal remedies in the United States and European countries [23]. Its popularity in the alternative medicine market may be attributed to its purported abilities to strengthen the immune system, inhibit inflammation, inhibit tumor growth, and suppress viral replication [24–26]. *Uncaria tomentosa* (*U. tomentosa*), one of the most common species of cat’s claw with higher alkaloid content than other species of cat’s claw, is preferred as raw material for Cat’s claw [23]. *U. tomentosa* has been known to possess extensive pharmacological activities, including anti-oxidative, anti-inflammatory, immune modulatory and anti-tumor activities [27]. Recent studies showed that *U. tomentosa* could alleviate the cognitive and memory impairments in both APP transgenic mice and middle-aged rats [28, 29]. However, the potency of the anti-AD effect of *U. rhynchophylla* and *U. tomentosa* has not been compared so far. Moreover, the molecular mechanisms underlying the neuroprotective effects of *U. tomentosa* still remains unexplored. *U. rhynchophylla* is used interchangeably with other *Uncaria* species, such as *U. tomentosa*, in clinical application, because of the similarity in chemical constituents. Thus, in the present study, we aimed to compare the chemical constituents and anti-AD activity of *U. rhynchophylla* and *U. tomentosa*.

## Methods

### Chemicals and reagents

Donepezil hydrochloride was purchased from Sigma-Aldrich (St. Louis, MO, USA). Streptozotocin (STZ, purity ≥ 98%) was purchased from Santa Cruz Biotechnology (Dallas, USA). Ethanol was obtained from DAEJUNG Chemicals (Gyeonggi-do, Korea). Methanol (HPLC grade) was bought from Duksan Pure Chemicals (Gyeonggi-do, Korea). Triethylamine (HPLC grade) was purchased from Scharlau (Barcelona, Spain). Standard substances (rhynchophylline, isorhynchophylline, corynoxeine and isocorynoxeine, purity ≥ 98%) were purchased from Chengdu Mansite Pharmacetical Co. Ltd (Chengdu, China). All other chemicals and reagents used were of analytical grade.

### Extraction and quality control of herbal materials

The *Uncariae Ramulus Cum Uncis* originated from the dried stem with hooks of *Uncaria rhynchophylla* was purchased from Zhixin Pharmaceutical Co., Ltd, a GMP-accredited Chinese herbal supplier based in Guangzhou, Guangdong Province, China. *Uncaria tomentosa* was purchased from Hking Bio-Tech Co., Ltd (Changsha, Hunan Province, China). They were authenticated to be the dried stem with hooks of *Uncaria rhynchophylla* (Miq.) Jacks. and *Uncaria tomentosa*, respectively, by Ms. Y. Y. Zong, a seasoned pharmacognist at the School of Chinese Medicine, The Chinese University of Hong Kong, where voucher specimens (No. 091220 and 091221, respectively) were deposited. For each raw herb, the dried herb was ground to powder or pieces. The herb (1000 g) was macerated in 10 L of 70% aqueous ethanol for 24 h at room temperature, and then extracted in an ultrasonic bath for 1 h. Extractions were repeated twice. Following filtration, the crude extracts were centrifuged to remove undissolved particles. The pooled extracts were combined and concentrated under reduced pressure, followed by freeze drying. The yields of the extracts of *U. tomentosa* (UTE) and *U. rhynchophylla* (URE) were 14.62% and 13.88%, respectively. The dried aqueous extracts were stored in desiccators at room temperature and avoid sunlight.

The high-performance liquid chromatography (HPLC) profiles of UTE and URE were constructed using an ACQUITY UPLC system (Waters, USA) equipped with a PDA eλ detector, a FTN sample manager, and quaternary solvent manager. Briefly, UTE or URE was dissolved in methanol and injected into UPLC by an autosampler. A Nucleosil 100 C18 HPLC column (4.6 mm × 250 mm, 5 μm) was used for separation. The mobile phase consisted of 0.01 mmol/L triethylamine in water (solvent A) and methanol (solvent B). Separation was achieved by a linear gradient elution from 60 to 85% solvent B over 40 min at a flow rate of 1.0 mL/min. The separation temperature was set at room temperature (25 °C) and detection wavelength was at 245 nm. The sample injection volume was 10 μL.

Method validation was performed based on a published procedure [30]. Calibration curves were established by plotting the peak area versus concentration of each analyte using a least-squares linear regression analysis. Limits of detection (LOD) and limits of quantification (LOQ) for each analyte were defined as signal-to-noise ratios (S/N) of 3 and 10, respectively. The precisions were determined by analyzing the five replicates of reference substances on the same day. Recovery tests were performed by spiking the raw material with reference standards at three levels, and then subjecting the solutions to the same sample preparation. Three replicates were performed for each analysis. To confirm the repeatability, five replicates of the same sample (UTE or URE) were analyzed. Variations were expressed in terms of relative standard deviation (RSD) in all of the tests.

### Animals

Adult male Sprague–Dawley (SD) rats weighing 230–250 g were obtained from the Laboratory Animal Services Centre, The Chinese University of Hong KONG. Rats were housed in controlled conditions (5 rats per cage, 12 h light/dark cycle with a constant room temperature of 20–24 °C and relative humidity of 40–60%) and had free access to food and water. This study was conducted in accordance with the Guide for the Care and Use of Laboratory Animals issued by the National Institutes of Health (NIH Publication No. 85-23, revised 2011). Approval for the experimental protocol was obtained from the Animal Experimentation Ethics Committee of The Chinese University of Hong Kong (Ref. No.18/264/MIS). The utmost possible efforts were made to minimize the number of animals used for this study and the experiment-induced suffering of the experimental animals.

### STZ-induced AD model

AD model was induced by intracerebroventricular (ICV) injection of STZ as previously described [14] with minor modifications. Briefly, rats were fixed on the stereotaxic apparatus (Stoelting, Wood Dale, IL, USA) under anesthesia with an intraperitoneal injection of ketamine (75 mg/kg) and xylazine (10 mg/kg). After a midline sagittal incision was made in the scalp, small burr holes (1 mm in diameter) were drilled. STZ (2 mg/kg, in a volume of 2 μL/ventricle, dissolved in 0.05 M citrate buffer, pH 4.5) was bilaterally injected into the lateral ventricle at a rate of 1 μL/min. The stereotaxic coordinates for ICV injection were as follows: 0.8 mm posterior to bregma, ± 1.4 mm lateral to sagittal suture and 4.0 mm ventral from the surface of the skull [31]. The sham-operated rats were subjected to the same surgical procedures but injected with the same volume of vehicle (0.05 M citrate buffer, pH 4.5). Rats had free access to food and water after recovery from anesthesia. Each rat received ICV injection of STZ or vehicle twice (on day 1 and day 3, respectively).

### Animal grouping and drug administration

Rats were randomly divided into five groups (n = 10): Sham group, STZ + vehicle (STZ-treated control) group, STZ + UTE (400 mg/kg) group, STZ + URE (400 mg/kg) group, and STZ + Donepezil (5 mg/kg) group. Donepezil was used as the positive control as described previously [20–22]. Drug treatment was initiated at one day after the second STZ injection. UTE, URE and donepezil hydrochloride were suspended in ddH_2_O, and administered intragastrically once daily for 6 consecutive weeks, while the same volume of the ddH_2_O was given to the rats in the Sham group and the STZ-treated group for the same duration.

### Morris water maze (MWM) test

The experimental apparatus consisted of a circular pool (150 cm in diameter, 45 cm in height), filled to a depth of 30 cm with water at 24 °C. The pool was conceptually divided into four equal quadrants for descriptive data collection. A circular escape platform (10 cm in diameter) was submerged 1 cm below the water surface in the midpoint of one quadrant (target quadrant). Some different geometric cues were equipped surrounding the pool, which can be used by experimental animals to determine the platform’s location. Rats were trained to find the hidden platform with 3 trials per day for four consecutive days. Each rat was placed in the water facing the pool wall from different release positions. The escape latency to climb onto the platform of each trail was recorded. The maximum trial time was 60 s. If a rat failed to find the platform within 60 s, it was manually guided to the platform and allowed to stay on it for 10 s (except for 30 s on the first day), and the escape latency was recorded as 60 s. The probe test was conducted without the hidden platform 24 h after the last training day. Rats were allowed to swim freely in the water for 60 s. A computerized video tracking system (SuperMaze V2.0 software, Shanghai Xinruan Information Technology Co., Ltd., China) was used to record the swimming paths, the numbers of the target quadrant crossing, the time spent in the target quadrant, and the swimming speed.

### Preparation of brain tissue samples

Twenty-four hours after the MWM test, rats were sacrificed under deep anesthesia. Then the rats were transcardially perfused with ice-cold saline (0.9%) until the fluid exiting the right atrium was entirely clear to remove peripheral blood from the CNS vasculature. The hippocampal tissues were rapidly isolated from the brains on ice and were stored at − 80 °C until use.

### Measurement of cytokines

Hippocampal tissues were homogenized in lysis buffer (Abcam) to prepare 10% (w/v) homogenates. The homogenates were centrifuged at 10,000*g* for 15 min at 4 °C. The supernatants were collected for assay and then for protein determination using a BCA Protein Assay kit (Invitrogen, USA). The levels of interleukin-1 β (IL-1β), IL-6 and tumor necrosis factor alpha (TNF-α) in the hippocampal tissues were measured using commercially available enzyme-linked immunosorbent assay (ELISA) kits (IL-1β and TNF-α from Abcam; IL-6 from RayBiotech) according to the manufacturer’s instructions.

### Measurement of the levels of superoxide dismutases (SOD), catalase (CAT), malondialdehyde (MDA) and glutathione peroxidase (GPx) activities

To evaluate the effects of UTE and URE on oxidative stress in the STZ-induced AD model, the activity levels of SOD, CAT, MDA and GPx were assessed. For these biochemical analyses, the brain tissues were homogenized and measured using SOD, CAT, MDA and GPx assay kits (Cat. No. 706002, 707002, 10009055 and 703102, respectively, Cayman, USA) following the manufacturer’s protocols. All samples were measured in duplicate.

### Western blot analysis

Approximately 20 mg of the hippocampus tissues were lysed in pre-chilled radio-immune precipitation assay buffer (RIPA; Invitrogen, USA) with a protease inhibitor cocktail (Sigma-Aldrich, USA) to prepare 10% (w/v) brain homogenates. The homogenates were centrifuged at 13,000 rmp for 20 min at 4 °C, and the supernatants were collected. The concentrations of proteins were determined using BCA Protein Assay kit (Invitrogen, USA). Equal amounts (30 μg) of denatured protein samples were separated on 10% SDS-polyacrylamide gels (SDS-PAGE), and transferred to polyvinyl difluoride (PVDF) membranes. After being blocked with 5% non-fat dry milk for 2 h, the membranes were rinsed and incubated overnight at 4 °C with primary antibodies, including mouse anti-Tau (Tau 46) antibody (1:500, Santa Cruz, sc-32274), mouse anti-Tau (Tau 5) antibody (1:500, Santa Cruz, sc-58860), rabbit anti-p-Tau (Thr181) antibody (1:1000, Cell Signaling, 12885S), rabbit anti-p-Tau (Ser396) antibody (1:10,000, Abcam, ab109390), rabbit anti-p-Tau (Ser404) antibody (1:1000, Abcam, ab92676), rabbit anti-Keap1 antibody (1:500, Santa Cruz), rabbit anti-Nrf2 antibody (1:1000, Abcam, ab31163), mouse anti-HO1 antibody (1:1000, Abcam, ab13248), rabbit anti-Akt antibody (1:1000, Cell Signaling, 4691P), rabbit anti-p-Akt (Ser473) antibody (1:1000, Cell Signaling, 4060S), rabbit anti-GSK-3β antibody (1:500, Santa Cruz, sc-9166), rabbit anti-p-GSK-3β (Ser9) antibody (1:1000, Cell Signaling, 9366S), rabbit Histone H3 antibody (1:500, Santa Cruz, sc-10809) and rabbit anti-GAPDH antibody (1:5000, Abcam, ab181602). Then, the membranes were treated with anti-rabbit IgG secondary antibody (1:3000, Cell Signaling, 7074S) or anti-mouse IgG secondary antibody (1:3000, Cell Signaling, 7076S) for 2 h at room temperature. The protein bands were visualized using enhanced chemiluminescence (ECL; Invitrogen, WP20005) reagent. Densitometric analysis was performed using Image J software.

### Statistical analysis

All data were presented as means ± standard deviation (mean ± SD) and all statistical analyses were performed using SPSS 20.0 software (SPSS Inc, Chicago, IL, USA). Group differences in the escape latency in the MWM training task were analyzed using two-way analysis of variance (ANOVA) with repeated measures, with the factors being treatment and training day. The other differences between multiple groups were analyzed by One-way ANOVA and differences between two groups were analyzed using t-test. A difference was considered statistically significant when *p* < 0.05.

## Results

### HPLC profile of UTE and URE

**Fig. 1:**
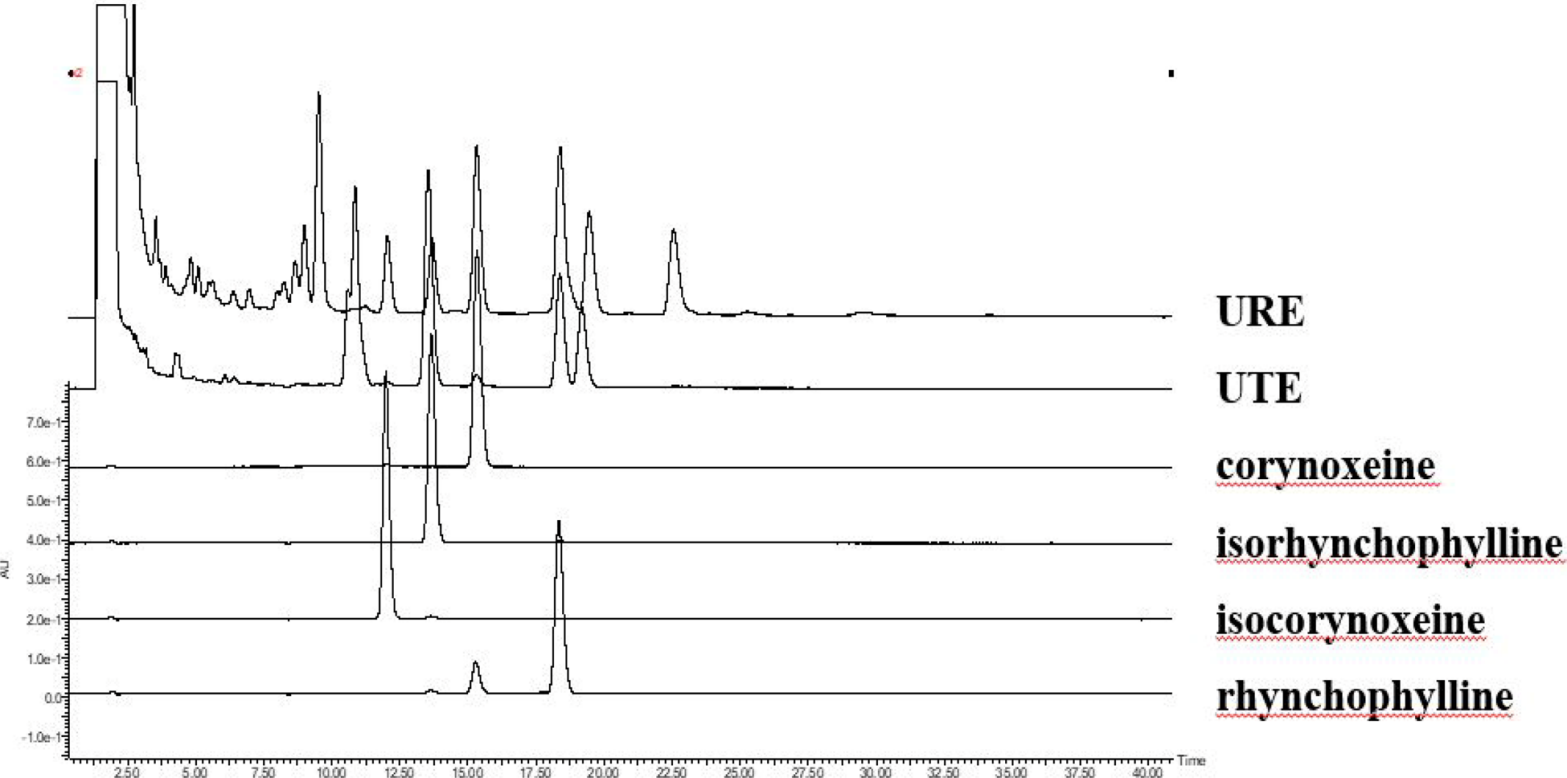
High-performance liquid chromatography profiles of 70% aqueous ethanol extracts of *Uncaria tomentosa* (UTE) and *Uncaria rhynchophylla* (URE). 1: isorhynchophylline; 2: corynoxeine; 3: rhynchophylline; 4: isocorynoxeine

Four representative compounds such as corynoxeine, isocorynoxeine and rhynchophylline and isorhynchophylline were used as chemical markers for the quality control of UTE and URE. Good linear relationships for all compounds were demonstrated (r^2^ ≥ 0.9995). Other HPLC method validation results including linear ranges, LOD, LOQ, precisions, repeatabilities, stabilities, and recoveries were shown in Table 1. Based on the calculation on the calibration curves of standard substances, the contents of four representative compounds in UTE and URE were shown as follows: UTE contains 0.278% rhynchophylline, 0.531% isorhynchophylline, 0.010% corynoxeine, and 0.028% isocorynoxeine, respectively; on the other hand, URE contains 0.478% rhynchophylline, 0.163% isorhynchophylline, 0.178% corynoxeine, and 0.418% isocorynoxeine, respectively. The HPLC chromatogram profiles of UTE and URE were shown in Fig. 1.

**Table 1:** The HPLC method validation for simultaneous quantification of the four markers in UTE and URE

Markers	Regression equation	Linear ranges (μg/mL)	*r*^*2*^	LOD (μg/mL)	LOQ (μg/mL)	Precision (RSD%)	Repeatability (RSD%)		Stability (RSD%)		Recovery (UTE)		Recovery (URE)	
							UTE	URE	UTE	URE	Mean (%)	RSD (%)	Mean (%)	RSD (%)
Rhynchophylline	*y* = 211.5*x* − 252.6	10–1000	0.9998	1.91	9.15	2.77	2.79	1.75	2.05	1.14	100.98	1.50	97.98	1.73
Isorhynchophylline	*y* = 223.7*x* − 2403	10–1000	0.9995	2.09	10.26	1.62	1.95	2.31	1.32	2.15	99.27	1.35	99.30	0.57
Corynoxeine	*y* = 152.6*x* + 1399	10–1000	0.9997	1.83	9.07	1.83	2.46	2.71	2.09	1.62	100.63	1.45	102.84	0.73
Isocorynoxeine	*y* = 107.7*x* − 140.4	10–500	0.9999	2.03	10.22	2.54	1.80	2.57	1.05	2.04	101.62	1.46	103.19	1.30

*x*, concentration in μg/mL; *y*, peak area; *r*^*2*^, correlation coefficient of regression equations

### Effects of UTE and URE on the cognitive impairments in the STZ-induced AD rats

**Fig. 2:**
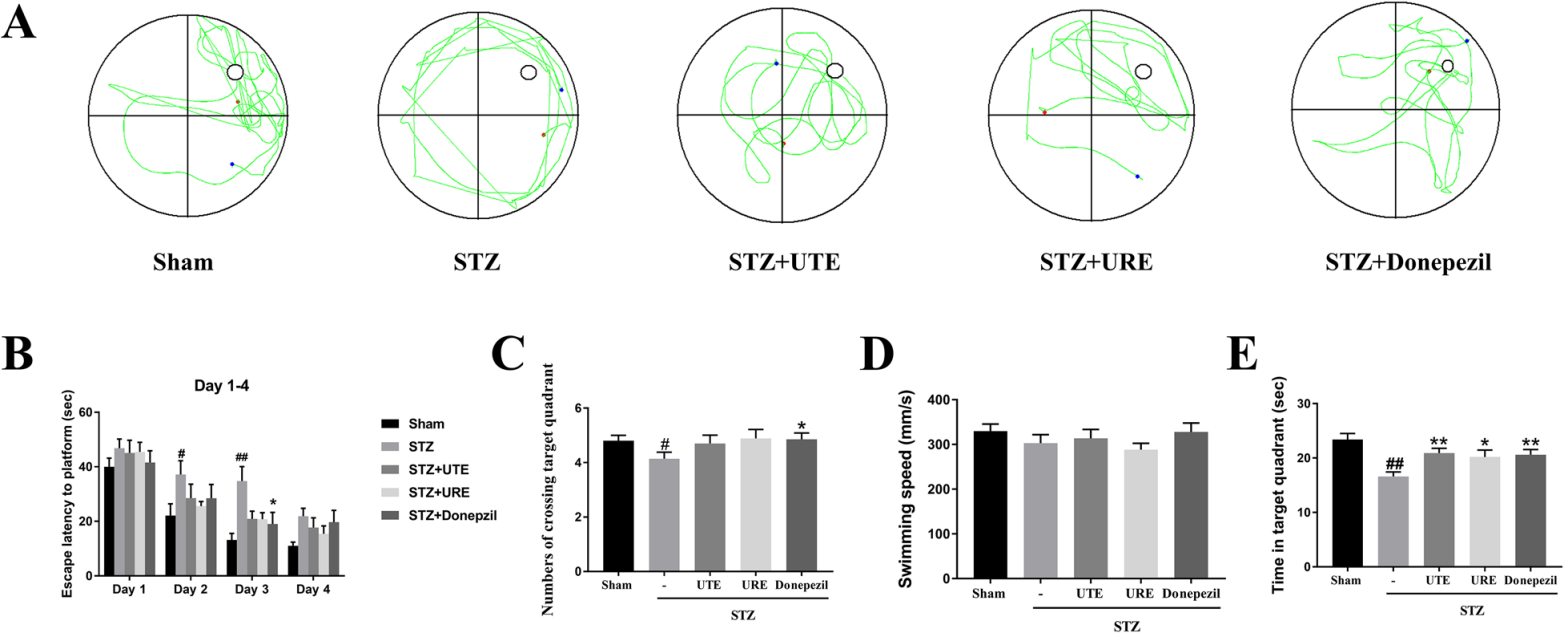
Effects of UTE and URE on the cognitive impairments in the STZ-induced AD rat model assessed by Morris Water Maze test (n = 9–10). **A** Representative images of the swimming path; **B** escape latency to platform during training days; **C** the number of target quadrant crossing in the probe tests; **D** swimming speed; **E** the time spent in the target quadrant in the probe test. Data were expressed as the mean ± SEM. ^#^*p* < 0.05 and ^##^*p* < 0.01 compared with the sham control group. *p < 0.05 and **p < 0.01 compared with the STZ control group

MWM test was used to determine learning and spatial memory in the STZ-induced AD rat model. The rats in the STZ + vehicle group had a longer escape latency to find the hidden platform than the Sham group during the training days (day 2, *p* < 0.05; day 3, *p* < 0.01), indicating that STZ treatment caused cognitive impairment to the rats. Compared with the STZ + vehicle group, the rats in both STZ + UTE group and STZ + URE group took a shorter time to find the platform but without statistically significant difference during the training days (from day 2 to day 4). In the probe trial, when compared with the Sham group, there was conspicuous reductions in the number of crossing target quadrant and time spent in the target quadrant in the STZ-treated group (*p* < 0.05 and *p* < 0.01, respectively), which was significantly reversed by the treatment of Donepezil. The STZ treated rats with the administration of both UTE and URE markedly spent more time in the target quadrant than vehicle treatment (*p* < 0.01 and *p* < 0.05, respectively). No statistical differences were observed in the swimming speed among all groups. These experimental findings indicated that both UTE and URE could ameliorate cognitive impairments in the STZ-induced AD rats (Fig. 2).

### Effects of UTE and URE on Tau pathology in the STZ-induced AD rats

**Fig. 3:**
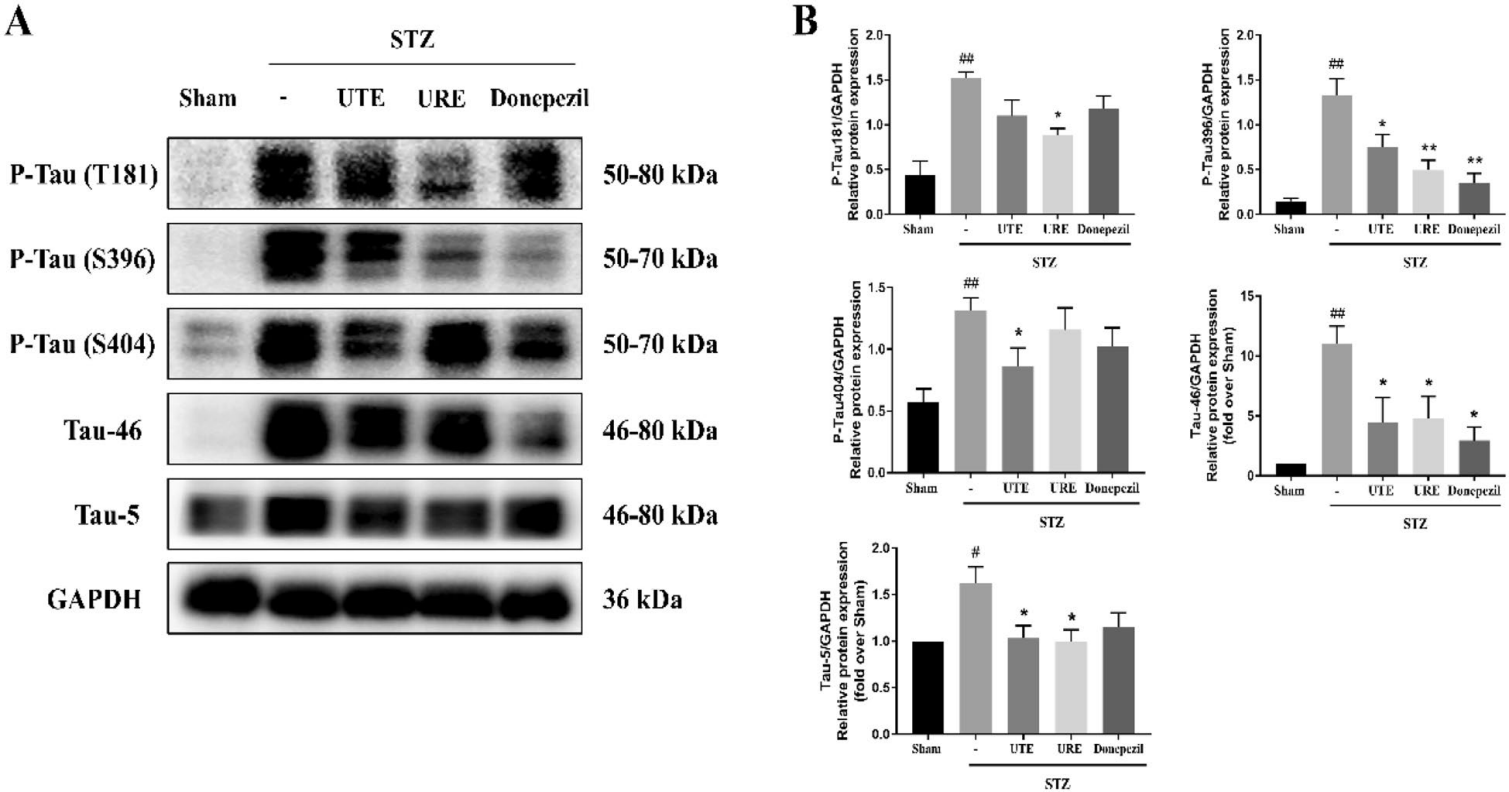
Effects of UTE and URE on tau hyperphosphorylation on the STZ-induced AD rat model (n = 5). Data were expressed as the mean ± SEM. ^#^*p* < 0.05 and ^##^*p* < 0.01 compared with the sham control group. *p < 0.05 and **p < 0.01 compared with the STZ control group

The protein expressions of total Tau (Tau-46 and Tau-5) in the STZ + vehicle group were significantly increased when compared with the Sham group (*p* < 0.01). Both of the STZ + UTE group and STZ + URE group had statistically lower protein expressions of total Tau than the STZ + vehicle group (*p* < 0.05 for both). Donepezil treatment (5 mg/kg) could suppress the level of Tau-46 when compared with the STZ-treated control group. Moreover, the protein levels of p-Tau (T181), p-Tau (S396) and p-Tau (S404) in the STZ control group were remarkably elevated (*p* < 0.01) when compared with the Sham group. UTE treatment could inhibit the hyperphosphorylation of Tau protein at the site of S396 (*p* < 0.05) and S404 (*p* < 0.05) when compared with the STZ control group, while URE treatment could reverse the protein expressions of p-Tau (T181) (*p* < 0.05) and p-Tau (S396) (*p* < 0.01). Similarly, Donepezil treatment (5 mg/kg) efficiently reduced the protein expression of p-Tau (S396) (*p* < 0.01), when compared with the STZ control group (Fig. 3).

### Effects of UTE and URE on the expression levels of pro-inflammatory cytokines in the STZ-induced AD rats

**Fig. 4:**
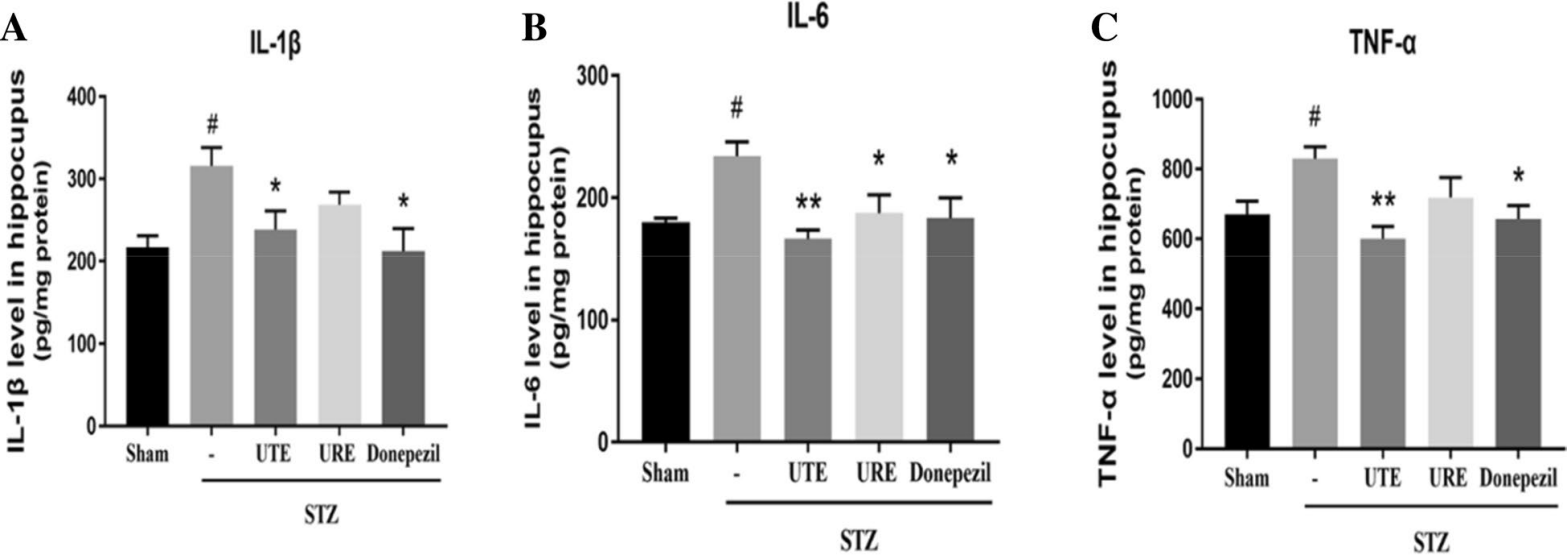
Effects of UTE and URE on pro-inflammatory cytokines in the STZ-induced AD rat model (n = 5). Data were expressed as the mean ± SEM. ^#^*p* < 0.05 and ^##^*p* < 0.01 compared with the sham control group. *p < 0.05 and **p < 0.01 compared with the STZ control group

Compared with the Sham group, there was a significantly enhanced levels of IL-1β, IL-6 and TNF-α in the STZ + vehicle group (*p* < 0.01). Treatment with UTE or Donepezil efficiently suppressed the elevated levels of IL-1β (*p* < 0.05 for both), IL-6 (*p* < 0.01 and *p* < 0.05, respectively) and TNF-α (*p* < 0.01 and *p* < 0.05, respectively) when compared with the STZ control group. URE treatment markedly inhibited the level of IL-6 (*p* < 0.05) and decreased the levels of IL-1β and TNF-α but without statistically significant difference, as compared with the STZ control group (Fig. 4).

### Effects of UTE and URE on oxidative stress in the STZ-induced AD rats

**Fig. 5:**
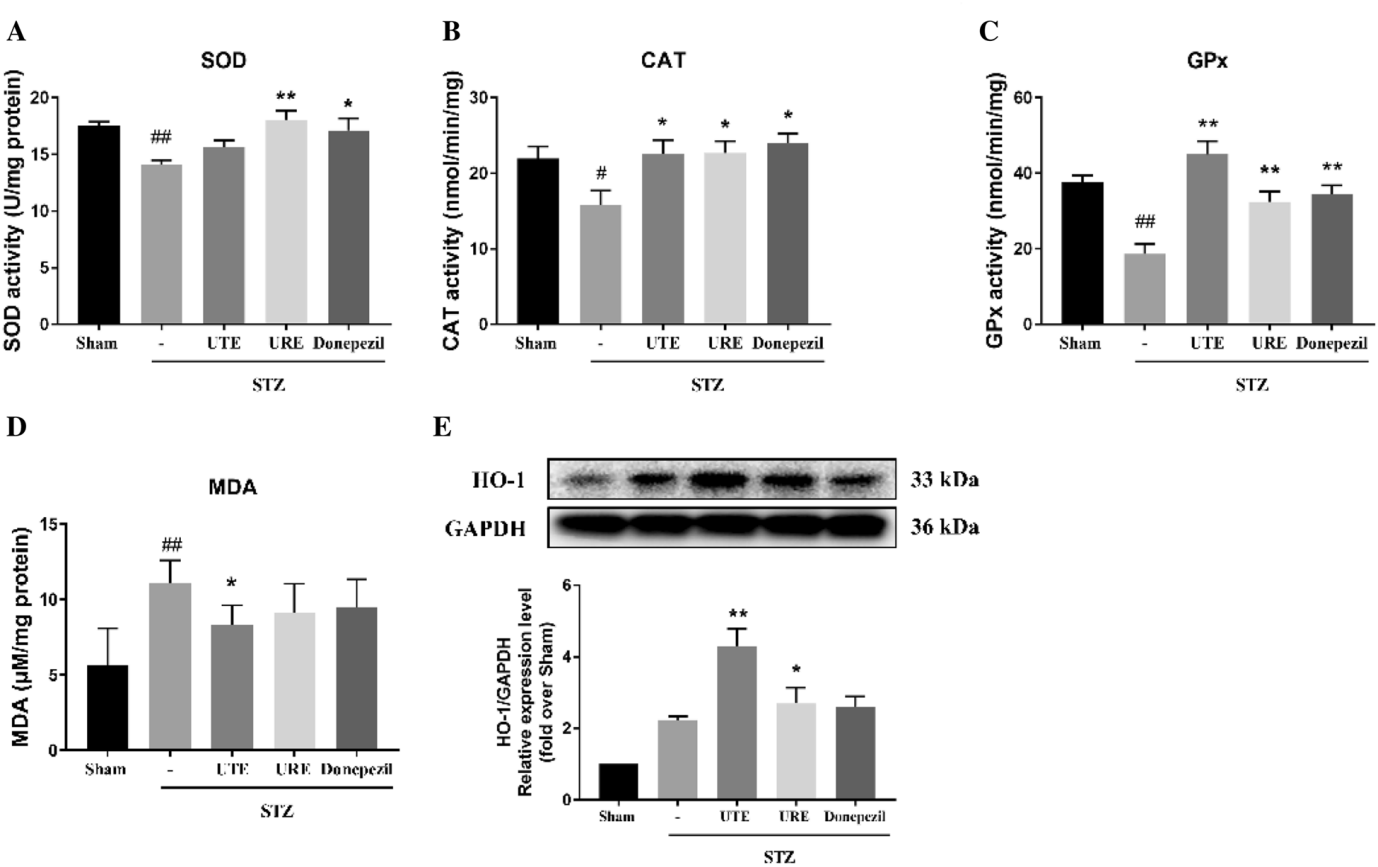
Effects of UTE and URE on oxidative stress in the STZ-induced AD rat model (n = 5). **A** SOD activity; **B** CAT activity; **C** GPx activity; **D** MDA level; **E** The protein expression of HO-1. Data were expressed as the mean ± SEM. ^#^*p* < 0.05 and ^##^*p* < 0.01 compared with the sham control group. *p < 0.05 and **p < 0.01 compared with the STZ control group

To assess the antioxidant potential of UTE and URE in the STZ-induced AD rats, the levels of oxidative damage marker (MDA), the antioxidant enzyme activities (SOD, CAT and GPx) and the protein expression of HO-1were measured. The SOD activity significantly decreased in the STZ + vehicle group (*p* < 0.01) as compared with the Sham group. Both URE and Donepezil treatment markedly (*p* < 0.01 and *p* < 0.05, respectively) increased the SOD activities as compared with the STZ + vehicle group. The STZ + UTE group had a higher level of SOD activity than the STZ + vehicle group, but no significant difference was found between the two groups (Fig. 5A).

The STZ + vehicle group had markedly lower CAT activity than the Sham group (*p* < 0.05). Compared with the STZ control group, all the treatment groups, including the STZ + UTE group, the STZ + URE group and the STZ + Donepezil group, significantly increased the levels of CAT activity (*p* < 0.05) (Fig. 5B).

The GPx activity was significantly reduced in the STZ control group (*p* < 0.01) as compared with the Sham group. After treatment, all the treatment groups, including the STZ + UTE group, the STZ + URE group and the STZ + Donepezil group, significantly increased the GPx activity as compared with the STZ control group (*p* < 0.01) (Fig. 5C).

When compared with the Sham group, the level of MDA, an oxidative damage marker, markedly accentuated in the STZ control group (*p* < 0.01). UTE treatment markedly reduced the MDA level as compared with the STZ control group (*p* < 0.05). In addition, both the STZ + URE group and the STZ + Donepezil group had lower MDA level than the STZ control group, but the differences were not significantly different (Fig. 5D).

### Effects of UTE and URE on the expression of HO-1 in the STZ-induced AD rats

The antioxidant effects of UTE and URE were further determined by analyzing the protein expression of HO-1 in the STZ-induced AD rats. There was no difference in the expression of HO-1 between the Sham group and the STZ control group. UTE and URE treatment significantly upregulated the HO-1 protein expression in the brains of the STZ-treated rats (*p* < 0.01 and *p* < 0.05, respectively) (Fig. 5E).

### Effects of UTE and URE on the protein expressions of Keap1 and nuclear Nrf2 in the STZ-induced AD rats

**Fig. 6:**
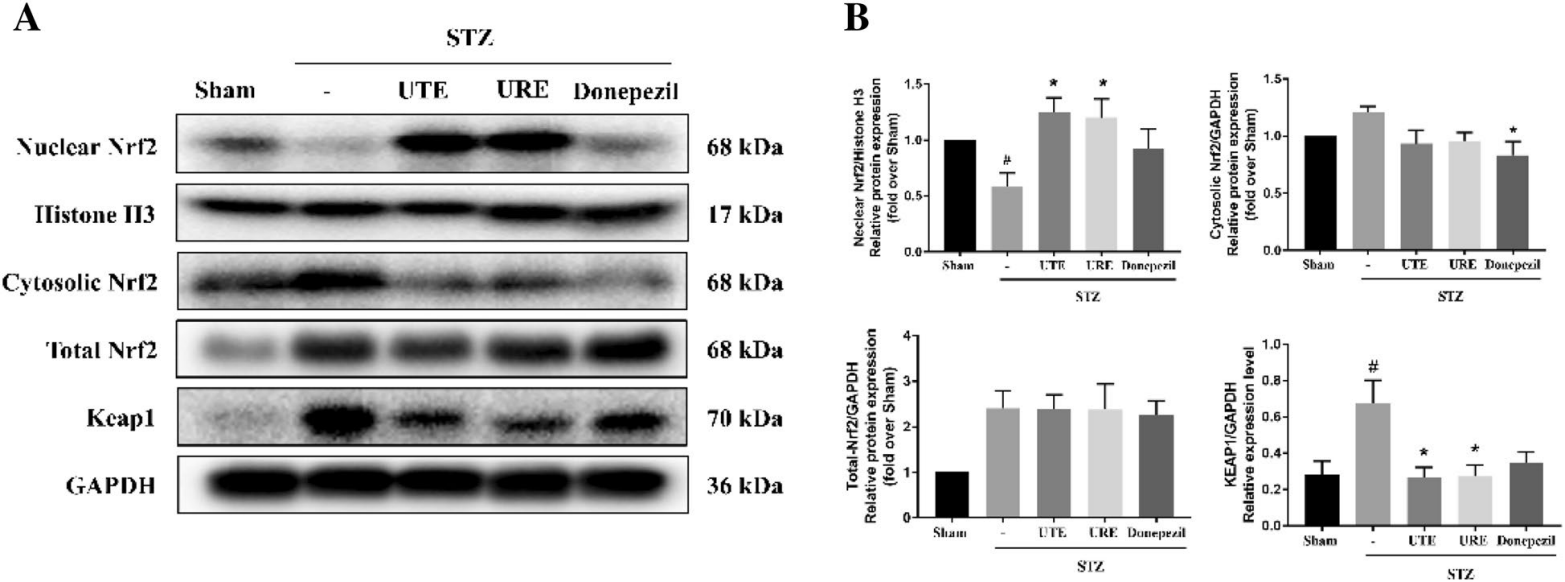
Effects of UTE and URE on the activated Nrf2 signaling in the STZ-induced AD rat model (n = 4–5). **A** The protein expressions of nuclear Nrf2, cytosolic Nrf2, total Nrf2 and Keap1; **B** Quantitative analysis for the western blot results. Data were expressed as the mean ± SEM. ^#^*p* < 0.05 and ^##^*p* < 0.01 compared with the sham control group. * *p* < 0.05 and **p < 0.01 compared with the STZ control group

The effects of UTE and URE on the nuclear translocation of Nrf2 were assessed by analyzing the protein expression of Nrf2 and its upstream target Keap1 in the STZ-induced AD rats. The western blot results showed that compared with the Sham group, there was a reduction in the protein expression of nuclear Nrf2 in the STZ control group (*p* < 0.05), and the reduction was reversed by UTE and URE treatment (*p* < 0.05 for both). In addition, both UTE and URE decreased cytoplasmic Nrf2 protein expression when compared with the Sham group, but the difference was not significant (*p* > 0.05 for both). STZ injection resulted in an increase in the protein expression of Keap1 when compared with the Sham group (*p* < 0.05), which was reduced upon UTE and URE treatment (*p* < 0.05 for both) (Fig. 6).

### Effects of UTE and URE on Akt/GSK-3β pathway in the STZ-induced AD rats

**Fig. 7:**
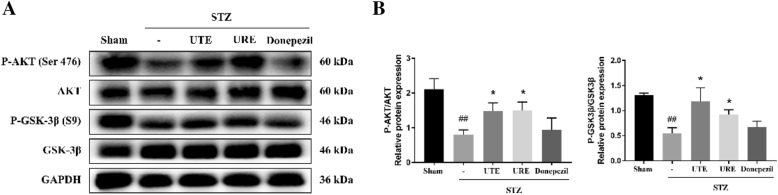
Effects of UTE and URE on Akt/GSK3β pathway in the STZ-induced AD rat model (n = 4–5). **A** The protein expressions of p-Akt (S473), Akt, p-GSK-3β (S9), and GSK-3β; **B** Quantitative analysis for the western blot results. Data were expressed as the mean ± SEM. ^#^*p* < 0.05 and ^##^*p* < 0.01 compared with the sham control group. *p < 0.05 and **p < 0.01 compared with the STZ control group

The western blot results showed that there were marked reductions in the relative ratios of p-Akt/AKT and p-GSK-3β/GSK-3β in the STZ control group (*p* < 0.01 for both), as compared with the Sham group. Both UTE and URE treatment effectively increased the relative ratio of p-Akt/AKT when compared with the STZ control group (*p* < 0.05 for both). Similarly, both UTE and URE treatment effectively increased the relative ratio of p-GSK-3β/GSK-3β when compared with STZ control group (*p* < 0.05 for both) (Fig. 7).

## Discussion

The present study demonstrated that both UTE and URE could significantly improve the cognitive and memory deficits via alleviating neuroinflammation, rescuing oxidative damage and inhibiting tau hyperphosphorylation through modulating Akt/GSK-3β pathway and Nrf2 activation-translocation.

The genus of *Uncaria*, belonging to the Rubiaceae family, consists of about 40 species all over the world, including *U. rhynchophylla* and *U. tomentosa*. More than 200 compounds have been isolated from the *Uncaria* plants up to now, of which the alkaloids exhibit marked pharmacological activities and have been regarded as the active chemical components for the treatment of AD [32–34]. Among these alkaloids, rhynchophylline (RN), isorhynchophylline (IRN), corynoxeine (CX), and isocorynoxeine (ICX) have been extensively studied, while other alkaloids derived from these *Uncaria* species have not been systematically studied yet [27, 35]. It was reported that RN, IRN, CX and ICX have the potent inhibitory activity against NO release from lipopolysaccharide (LPS)-activated microglia [36]. Our previous study has demonstrated that IRN (20 mg/kg or 40 mg/kg) exerted neuroprotective effects and ameliorated cognitive impairments in several animal models of AD, including Aβ_25–35_-induced rats, D-galactose-induced mice and transgenic APP mice [20–22], indicating that IRN is a therapeutic candidate in the treatment of AD. Moreover, our in vitro experiments showed that both IRN and RN markedly decreased the Aβ-induced neurotoxicity and apoptosis in PC12 neuronal cells [18, 19, 37]. RN was also found to improve the Aβ_1-42_-induced spatial cognitive impairments in rats [38] and suppress the Aβ-induced enhancement of spontaneous discharges in the hippocampal CA1 region of rats [39], suggesting that RN has neuroprotective effects on AD. It has been reported that 5 alkaloids in *U. rhynchophylla* including IRN and ICX are likely to be vital components for AD treatment in a potential alkaloid target-AD target network using a network pharmacology approach [33]. RN, IRN CX, and ICX are representative indole alkaloids from *U. rhynchophylla* and *U. tomentosa*. Our HPLC analysis results showed that URE contained 0.478% RN, 0.163% IRN, 0.178% CX, and 0.418% ICX, and the results were generally congruent with our previous study [17]. UTE was found to be enriched with RN and IRN (0.278% and 0.531%, respectively) but have much less CX and ICX (0.010% and 0.028%, respectively) than URE. Although the contents of UTE and URE varied, both UTE and URE could effectively ameliorate the cognitive and memory deficits in the STZ-induced rats, demonstrating that the protective effects of UTE and URE may be attributed to the synergistic effects of their predominant active component alkaloids such as RN and IRN.

An ideal animal model of AD is indispensable to evaluate the potential efficacy of a therapy, and to explore its underlying anti-AD molecular mechanisms. The inappropriate choice of preclinical AD animal models might result in the high failure rate of drug development in clinical trials [40]. As we know, AD can be divided into familial AD (fAD) and sporadic AD (sAD). Although fAD is much less common (accounting for only 5% of all AD cases), many anti-AD drugs under clinical development were evaluated based on the fAD animal models, in which the underlying mechanisms are primarily associated with familial mutations [40, 41]. Patients diagnosed with sAD make up over 95% of all cases and have no documented familial history of AD [42, 43]. The present study adopted ICV administration of STZ in rats to establish sporadic AD model. STZ is a natural alkylating anti-neoplastic agent which is selectively toxic for insulin producing cells both in the periphery and in the brain. It has been well recognized as an appropriate sporadic AD model closely resembling AD in humans in terms of progressive memory deficits, abnormal cerebral glucose and energy metabolism, decreased expressions of insulin receptor (IR), induction of hyperphosphorylated tau as well as Aβ plaque deposition [44, 45]. In addition, Mehla et al*.* [46] found that the cognitive deficits induced by both single and twice ICV-STZ injections (3 mg/kg) persist from the 2nd week after STZ injection to the end of the 14-week experiment. In the present study, the MWM results showed that a significant decrease was found in the number of target quadrant crossing and time spent in the target quadrant in the STZ treated group in comparison with the sham group, indicating that STZ could cause cognitive impairments and the STZ-induced AD model was successfully established. In addition, we also found that STZ could induce many pathological features of AD, including neuroinflammation, oxidative stress and hyperphosphorylation of tau protein. Both UTE and URE treatment could ameliorate the cognitive deficits induced by STZ in rats.

Neuroinflammation has been well recognized as a prominent contributor in AD, involving in not only the onset, but also the pathological process of AD [47, 48]. The release of pro-inflammatory markers, such as IL-1β, IL-6 and TNF-α, can cause neuronal damage, synaptic dysfunction and inhibition of neurogenesis [49]. IL-1 is a major driver of inflammation which has a clear detrimental effect and causes neuronal injury in AD and other chronic neurodegenerative disorders [50, 51]. It has been indicated that IL-1β is closely associated with neurodegeneration and impaired learning and memory functions [52]. IL-1β is elevated in AD patients, along with its antagonist IL-1Ra and its soluble receptor sIL-1R1 [50]. Similarly, as the main pro-inflammatory cytokines in the brain, IL-6 and TNF-α have been extensively investigated for their roles in AD pathology. Studies showed that the levels of IL-6 and TNF-α were elevated in the brain and in cerebrospinal fluid (CSF) of AD patients, while IL-6 may be a predictor for the severity of cognitive defects [53, 54]. In line with these findings, the increased levels of these pro-inflammatory cytokines were also observed in the AD animal models [55]. Moreover, neuroinflammation and microglial activation can exacerbate tau pathology by promoting hyperphosphorylation and aggregation of tau protein [56]. Thus, anti-inflammatory intervention has been identified as a potential therapeutic approach for this disease [57]. The present study revealed that both UTE and URE exerted anti-neuroinflammatory action via reducing the elevated levels of IL-1β, IL-6 and TNF-α in the STZ-induced AD rat model.

Oxidative stress, characterized by an imbalance in reactive oxygen species (ROS) and anti-oxidative defense, is an early feature of AD and plays a critical role in AD pathogenesis [58]. The impairment of anti-oxidative systems in the AD brain leads to the decreased activities of antioxidant enzymes (e.g. SOD, CAT and GPx) and the increased generation of free radicals, thereby causing neuronal damage by promoting lipid peroxidation, protein breakdown and DNA damage [59]. Lipid peroxidation, mediated by superoxide, can directly contribute to the blood brain barrier (BBB) disruption, leading to the influx into the brain of inflammatory mediators and neurotoxic blood-derived debris, and these events subsequently initiate multiple mechanisms of neurodegeneration of AD [60]. In addition, the end products of lipid peroxidation, such as MDA and 4-hydroxy2-nonenal (4-HNE), are found to be increased in the AD brain [61]. Emerging evidence suggested that interventions targeting anti-oxidative bioavailability are potential therapeutic strategies for AD [62]. The present study demonstrated that both UTE and URE exerted anti-oxidative effects by enhancing the activities of antioxidant enzymes (SOD, CAT and GPx) and increasing the protein expression of HO-1. Moreover, UTE could decrease the MDA level.

Tau protein is of great importance for stabilizing microtubules under physiological conditions [63]. In the context of AD, the pathological modification of tau, i.e., hyperphosphorylated tau, detaches from microtubules, aggregates into paired helical filaments (PHFs) and forms neurotoxic NFTs, causing synaptic loss, neuronal dysfunction, and eventually cognitive impairment [64]. Thus, the progression of AD inevitably accompanies with abnormal hyperphosphorylation and aggregation of tau protein. Furthermore, hyperphosphorylated tau pathology correlates with symptom severity, and is more closely related to cognitive dysfunction than amyloid deposition in AD patients [65]. Overwhelming evidence demonstrated that there are prominently increased levels of phosphorylated tau (p-tau) at the sites of Thr181, Ser396 and Ser404 in AD patients [66]. Recent study revealed that p-tau Thr181 is a highly novel biomarker for AD diagnosis [67], while p-tau Ser396 and p-tau Ser404 are also found to play an important role in AD pathology [68]. The present study showed that UTE could reverse the elevated expressions of total tau, p-tau Ser396 and p-tau Ser404 while URE could reverse the elevated expressions of total tau, p-tau Ser181 and p-tau Ser396, indicating that suppressive effects of UTE and URE on these specific hyperphosphorylation sites of tau protein is one of the mechanisms underlying their cognitive improving properties.

Nrf2, the master regulator of homeostatic responses, is considered as a promising therapeutic target for AD and an essential component in modulation of oxidative stress and neuroinflammation in AD [69]. The Nrf2 transcriptional activity declines with age [70], and it was reported that not only nuclear Nrf2 levels, but also Nrf2-mediated transcription are suppressed in the hippocampus of AD patients [71]. Nrf2 deficiency worsens APP and Tau pathology and exacerbates cognitive deficits in preclinical AD models, which is associated with increased levels of oxidative and pro-inflammatory markers [72]. Conversely, up-regulation of Nrf2 by intra-hippocampal injection can restore spatial learning impairment in transgenic AD mice [73]. In an inactive state, the transcriptional role of Nrf2 is repressed, due to its binding to Kelch-like ECH associated protein 1 (Keap 1) in the cytoplasm [74]. Many studies demonstrated that inactive GSK-3β activity can alter the conformation of Keap1-Nrf2 complex and disrupt their interaction, thereby increasing Nrf2 nuclear translocation and Nrf2 related downstream antioxidant gene expression (such as HO-1, NQO-1 and CAT) [75, 76]. Unlike most cellular kinases that are normally active, GSK-3β is inactive under phosphorylation (Ser9) that is regulated by activated Akt. GSK-3β activation is elevated [77] and regulates tau phosphorylation [78] in AD patients. Conditional overexpression of GSK-3β causes neuronal death, hyperphosphorylation of tau protein and cognitive impairments in mice [79], while GSK-3β inhibitors have been found to reverse cognitive deficits in several rodent models of AD [80]. It has been demonstrated that the PI3K/Akt/GSK-3β pathway plays a pivotal role in the regulation of Nrf2 nuclear export and degradation in AD [81]. Recent studies targeted Akt/GSK-3β-mediated Nrf2 activation as a therapeutic potential in CNS diseases, such as AD [82] and ischemic stroke [83]. This study showed that increased protein expression of nuclear Nrf2 is accompanied with an increase of Akt activation (phosphorylation) and GSK3β inactivation (phosphorylation) after UTE and URE treatment in the STZ-induced AD model, indicating that Akt(Ser473)/GSK3β(Ser9) may mediate the neuroprotective effects of UTE and URE.

## Conclusion

**Fig. 8:**
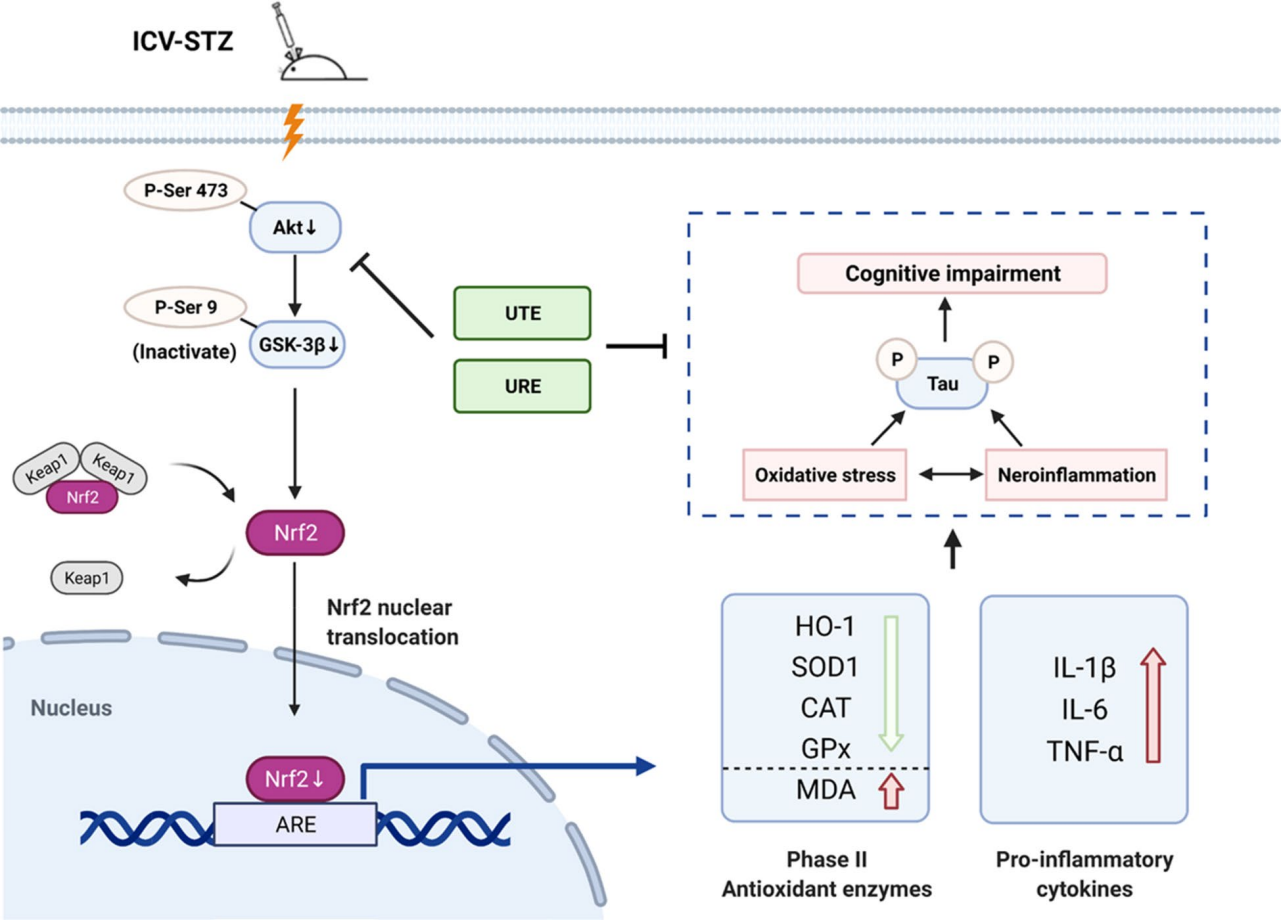
The schematic drawing summarizing the proposed mechanisms for the anti-neuroinflammatory and antioxidant effects of UTE and URE in the STZ-induced AD rat model. The mechanisms involve the inhibition of hyperphosphorylated tau protein, the promotion of antioxidant enzymes expression, the reduction of pro-inflammatory cytokines and the role of Akt (Ser473)/GSK3β (Ser9)-mediated Nrf2 activation

In this present study, we indicated for the first time that UTE and URE, the two herbal products with similar chemical constituents, could alleviate cognitive impairments via suppressing tau hyperphosphorylation in the STZ-induced AD rats, and the effects are largely attributed to their anti-oxidant and anti-neuroinflammatory activities via Akt (Ser473)/GSK3β (Ser9)-mediated Nrf2 activation (Fig. 8). Furthermore, this study provides preclinical evidence for the comparative effects of UTE and URE on AD, suggesting that both UTE and URE can be potential candidates for further clinical trials. Finally, the major bioactive components in UTE and URE are worthy of further investigation for the anti-AD effects.

## Data Availability

I agree to share my data and materials.

## Abbreviations

4-HNE: 4-Hydroxy2-nonenal
Aβ: Beta-amyloid
AChEIs: Acetylcholinesterase inhibitors
AD: Alzheimer’s disease
BBB: Blood brain barrier
CAT: Catalase
CSF: Cerebrospinal fluid
ECL: Enhanced chemiluminescence
GPx: Glutathione peroxidase
HPLC: High-performance liquid chromatography
ICV: Intracerebroventricular
IL-1β: Interleukin-1β
IRN: Isorhynchophylline
Keap1: Kelch-like ECH associated protein 1
MDA: Malondialdehyde
MWM: Morris water maze
NFTs: Neurofibrillary tangles
NMDA: *N*-Methyl-d-aspartate
PHFs: Paired helical filaments
PVDF: Polyvinyl difluoride
ROS: Reactive oxygen species
SD: Sprague–Dawley
SOD: Superoxide dismutases
STZ: Streptozotocin
TNF-α: Tumor necrosis factor alpha
UTE: Extract of *Uncaria* *tomentosa*
URE: Extract of *Uncaria* *rhynchophylla*
